# Case Report: NUTM1-rearranged lung sarcoma: a rare case imaged with dynamic and static ^18^F-FDG PET/CT

**DOI:** 10.3389/fonc.2025.1663104

**Published:** 2025-09-10

**Authors:** Xieraili Wumener, Xiaoxing Ye, Jiuhui Zhao, Yarong Zhang, Tuya E, Jun Zhao, Ying Liang

**Affiliations:** ^1^ Department of Nuclear Medicine, National Cancer Center/National Clinical Research Center for Cancer/Cancer Hospital & Shenzhen Hospital, Chinese Academy of Medical Sciences and Peking Union Medical College/Shenzhen Clinical Research Center for Cancer, Shenzhen, China; ^2^ Department of Pathology, National Cancer Center/National Clinical Research Center for Cancer/Cancer Hospital & Shenzhen Hospital, Chinese Academy of Medical Sciences and Peking Union Medical College/Shenzhen Clinical Research Center for Cancer, Shenzhen, China; ^3^ Department of Radiology, National Cancer Center/National Clinical Research Center for Cancer/Cancer Hospital & Shenzhen Hospital, Chinese Academy of Medical Sciences and Peking Union Medical College/Shenzhen Clinical Research Center for Cancer, Shenzhen, China; ^4^ Department of Nuclear Medicine, Shanghai East Hospital Tongji University, Shanghai, China

**Keywords:** NUTM1-rearranged lung sarcoma, 18F-FDG, PET/CT, dynamic, CT

## Abstract

**Background:**

Nuclear protein of testis midline carcinoma family member 1 (NUTM1)-rearranged lung sarcomas are rare malignant malignancies. ^18^F-fluorodeoxyglucose (FDG) positron emission tomography/CT (PET/CT) is widely used for the differential diagnosis and staging of malignant tumours. This study aimed to identify the static and dynamic features of ^18^F-FDG PET/CT in NUTM1-rearranged lung sarcomas.

Case Description: A 46-year-old male patient underwent a chest CT scan for trauma, which revealed a tumor in the hilum of the left lung. The patient also underwent dynamic (chest) and static (whole body) PET/CT scans. The dynamic and static ^18^F-FDG PET/CT scans showed a tumor in the hilum of the left lung, with a size of 8.6×6.7 cm, an SUV_max_ of 17.6 and a K_i_ of 0.0668 ml/g/min. Final pathology and genetic testing confirmed NUTM1-rearranged lung sarcoma.

**Conclusions:**

This case shows dynamic and static ^18^F-FDG PET/CT and pathological features of NUTM1-rearranged lung sarcomas.

## 1 Introduction

Nuclear protein of testis midline carcinoma family member 1 (NUTM1)-rearranged lung sarcomas are rare malignant malignancies (1). Currently, there are very few reports on NUTM1-rearranged lung sarcomas. The few reports that exist tend to be case studies focusing on pathological features (2, 3). The imaging features of NUTM1-rearranged lung sarcoma have been under-recognised.

^18^F-fluorodeoxyglucose (FDG) positron emission tomography/CT (PET/CT) has been widely used for diagnosing and staging tumours, as well as evaluating their efficacy and prognosis. In recent years, with the development of molecular imaging technology, the application of dynamic PET imaging in oncology has attracted much attention. Dynamic PET scans extract physiological parameters that reveal pathophysiological mechanisms of disease more effectively (4). Therefore, dynamic quantitative metabolic parameters (e.g., Ki) are potentially advantageous in tumor differential diagnosis, efficacy assessment, and prognostic evaluation (5–9).

In this study, we report the clinical, lung cancer-related serum tumor markers, CT scan and dynamic+static ^18^F-FDG PET/CT features of a patient diagnosed with NUTM1-rearranged sarcoma by pathological and genetic test results to deepen the understanding of the imaging features of this disease.

## 2 Case presentation

**Figure 1:**
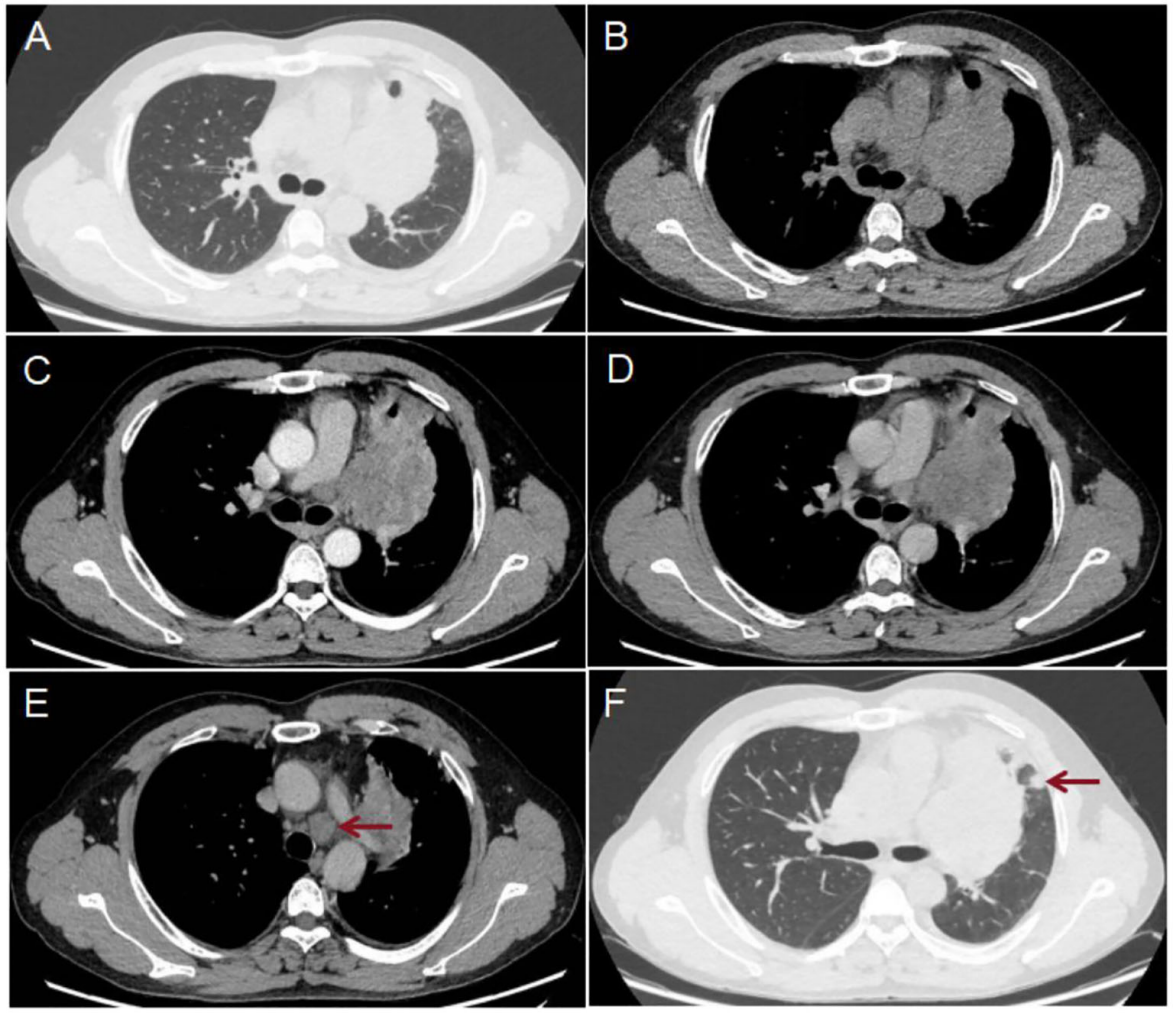
Results of the patient’s chest CT scan: The scan showed a lesion in the hilum of the left lung **(A, B)**, size of 10.8×7.6 cm. The enhanced scan [**(C)** arterial phase; **(D)** venous phase] shows moderate inhomogeneous enhancement. The largest LN was located in the 4L region of the mediastinum [**(E)** red arrow] and measured 2.5×1.8 cm. Several solid nodules were present in the upper lobe of the left lung, the largest of which was located subpleurally [**(F)** red arrow] and size of 1.0×0.9 cm.

A 46-year-old male patient underwent a chest CT scan for trauma. **Figure 1** shows the patient’s chest CT scan. The results of the chest CT scan showed a tumor in the hilum of the left lung, size of 10.8×7.6 cm. The tumor exhibited moderate inhomogeneous enhancement on the enhancement scan and encroached upon the mediastinum, mediastinal lymph nodes (LN), pulmonary arteries and veins, and the pericardium. Multiple LNs in the supraclavicular and mediastinal regions bilaterally, the largest LN was located in the 4L region of the mediastinum, with a size of 2.5×1.8 cm. There are several solid nodules in the upper lobe of the left lung. The largest is located in the subpleura,with a size of 1.0×0.9 cm. The chest CT scan results suggest lung cancer, accompanied by partial LNs metastases and nodules in the left upper lobe of the lung that are suggestive of metastasis. Lung cancer related serum tumor markers were negative, including carcinoembryonic antigen (CEA, 1.72 ng/ml, 0-5.0ng/ml), pro-gastrin-releasing peptide (Pro GRP, 27.90 pg/ml, <69.2pg/ml), recombinant cytokeratin fragment antigen 21-1 (CYFRA21-1, 1.87ng/ml, <3.3ng/ml), carbohydrate antigen 125 (CA125, 14.1 U/ml, <35.0 U/ml), neuron-specific enolase (NSE, 15.24 ng/ml, <16.3 ng/ml), and squamous cell carcinoma antigen (SCC, 1.28 ng/ml, <2.7 ng/ml).

**Figure 2:**
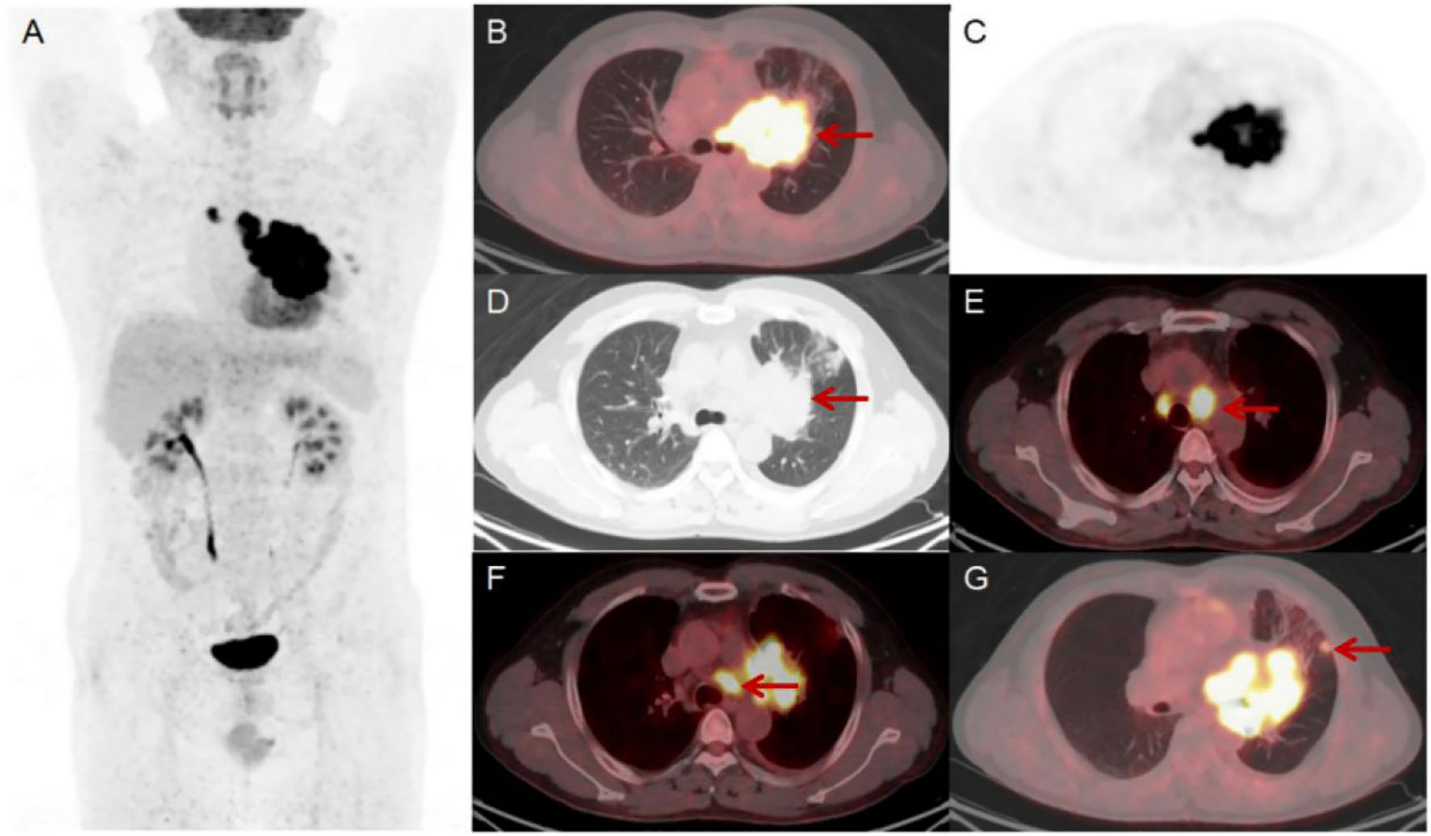
Results of the patient’s static ^18^F-FDG PET/CT scan **(A-G)**. The scan showed a lesion in the hilum of the left lung, size of 8.6×6.7 cm [**(D)** red arrow] with an SUV_max_ of 17.6 [**(B)** red arrow]. There were enlarged LNs in mediastinal regions 4R, 4L and 5 that were FDG-avid [**(E, F)** red arrow]. The largest LN was located in region 4L and size of 2.6×2.6 cm with an SUV_max_ of 14.9 [**(E)** red arrow]. Additionally, there were two FDG-avid nodules in the upper lobe of the left lung, the largest of which size of 1.2×1.1 cm with an SUV_max_ of 4.0 [**(G)** red arrow].

**Figure 3:**
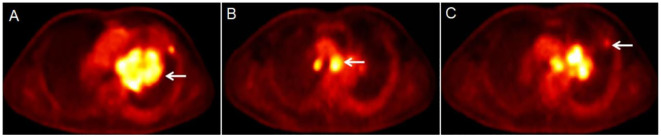
Results of the patient’s dynamic ^18^F-FDG PET/CT scan **(A-C)**. The dynamic ^18^F-FDG PET/CT scan showed a lesion in the hilum of the left lung [**(A)** white arrow], with a K_i_ of 0.0668 ml/g/min. The largest LN was located in region 4L [**(B)** white arrow], with a K_i_ of 0.0650 ml/g/min. The largest node was in the upper lobe of the left lung [**(C)** white arrow], with a K_i_ of 0.0183 ml/g/min.

The patient underwent a dynamic (chest, 65 min) + static (whole body, 10–20 min) ^18^F-FDG PET/CT scan for definitive diagnosis and pre-treatment staging. Before the ^18^F-FDG injection, the patient had fasted for at least 6 h and had a pre-scan glucose level of 6.1 mmol/L. PET scans of the chest region were initiated immediately after the injection of ^18^F-FDG (8.37 mCi) via an intravenous indwelling needle, according to the body mass index. The dynamic scans were carried out for a duration of 65 minutes. Dynamic scan data were then partitioned into 28 frames as follows: 6 × 10 s, 4 × 30 s, 4 × 60 s, 4 × 120 s, and 10 × 300 s. Quantitative parameters (K_i_) was obtained through applying the irreversible two-tissue compartment model using in-house Matlab software. A whole-body static PET/CT scan was performed at the end of the dynamic acquisition process. **Figures 2**, **3** show the patient’s dynamic and static ^18^F-FDG PET/CT scans of the patient. The dynamic and static ^18^F-FDG PET/CT scan showed a tumor in the hilum of the left lung (**Figures 2A-D**), with a size of 8.6×6.7cm (**Figure 2D**), an SUV_max_ of 17.6 (**Figure 2B**), and a K_i_ of 0.0668 ml/g/min (**Figure 3A**). Enlarged LNs in mediastinal regions 4R, 4L, and 5 with FDG-avid (**Figures 2E, F**), the largest LN was located in region 4L, with a size of 2.6×2.6 cm, an SUV_max_ of 14.9 (**Figure 2E**), and a K_i_ of 0.0650 ml/g/min (**Figure 3B**). Futhermore, two nodules in the upper lobe of the left lung with FDG-avid (**Figure 2G**), the largest size of 1.2×1.1 cm, with an SUV_max_ of 4.0 (**Figure 2G**), and a K_i_ of 0.0183 ml/g/min (**Figure 3C**).

**Figure 4:**
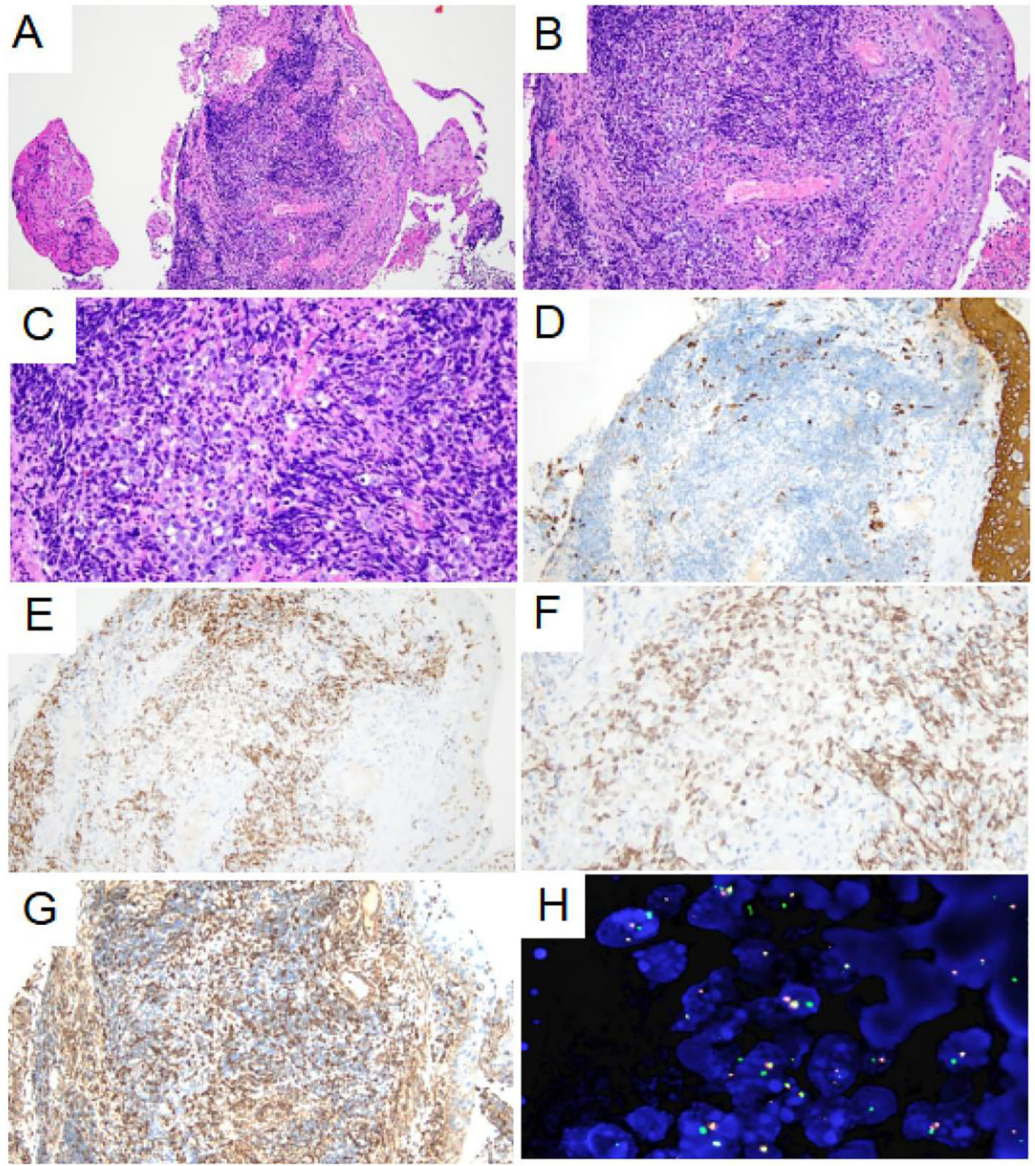
Pathological morphology and immunohistochemical staining of tumor. **(A)** Low-power histological examination (H&E stain, 100 ×) of bronchoscopy biopsy demonstrates that the tumor was located in the fibrous tissue of the bronchial mucosa. **(B)** At medium magnification, the tumor cells were distributed in sheet and nest shape, composed of monomorphic small round or short spindle cells, with little cytoplasm and most of the cytoplasm was transparent (H&E stain, 200 ×). **(C)** High-power of the tumor showed no obvious nuclear pleomorphism, fine chromatin, obvious nucleolus and mitoses are easily visible (H&E stain, 400 ×). **(D)** AE1/AE3 immunohistochemical staining showed negative expression of the tumor cell (200 ×). **(E-F)** NUT1 immunohistochemical staining showed moderately granular positive expression of tumor nucleus (200× and 400×). **(G)** VIM immunohistochemical staining showed weak positive expression of tumor cell membrane and para-nuclear (200 ×). **(H)** Fluorescence *in situ* hybridization detection showed the presence of NUTM1 gene separation (1000 ×).

The patient then underwent a bronchoscopic puncture biopsy. Final pathological and genetic test results confirmed NUTM1-rearranged lung sarcoma. **Figure 4** shows the pathological morphology, immunohistochemical staining, and genetic testing results.

## 3 Discussion

NUTM1-rearranged lung sarcomas can occur at any age and are gender-neutral, with an atypical clinical presentation, aggressive nature and poor prognosis (2, 3). NUTM1-rearranged sarcomas have been reported with pathological features in a variety of different soft tissue sites in the body, including the stomach, kidneys, brain, ovaries and gastrointestinal tract (10–13). Most reports are dominated by case reports of the pathological features of NUTM1-rearranged lung sarcoma have been under-recognised.

In this case, we describe the clinical, CT, dynamic and static ^18^F-FDG PET/CT scan features of a patient diagnosed with NUTM1-rearranged lung sarcoma, as confirmed by pathological and genetic test results, to further our understanding of the imaging features of this disease. This is a 46-year-old male patient with an incidental finding of a lung tumour and no associated clinical manifestations. The chest CT scan showed a tumor in the hilum of the left lung and moderate inhomogeneous enhancement on the enhancement scan and invasion of adjacent tissues, reflecting the malignancy and invasiveness of the tumor. In addition, refuinement lung cancer related serum tumor markers showed negative, including CEA, Pro GRP, CYFRA21-1, CA125, NSE, and SCC.

The patient’s static ^18^F-FDG PET/CT scan showed a tumor in the left lung and FDG-avid, an SUV_max_ of 17.6. The SUV_max_ value of the primary lesion was particularly suggestive of the malignancy of the disease. And the ^18^F-FDG PET/CT scan showed FDG-avid LNs in the mediastinal regions 4R, 4L, and 5 to help with accurate N staging. Previous reports have shown that the disease tends to be highly aggressive and may be prone to distant metastasis (2, 3). ^18^F-FDG PET/CT also help with precise M-staging, which is important for managing the disease. As NUTM1-rearranged lung sarcoma is so rare, there are currently no relevant studies on imaging-based differential diagnosis. We believe that the ^18^F-FDG PET/CT features of NUTM1-rearranged lung sarcoma resemble those of NUT carcinoma (14) and SMARCA4-deficient NSCLC (15). However, ^18^F-FDG PET/CT is still mainly used to assist in TNM staging in these diseases and cannot yet be used for differential diagnosis based on imaging features. A pathological diagnosis is still required for the final diagnosis. In this case, ^18^F-FDG PET/CT scan ruled out distant metastases, helping clinicians to accurately stage patients and develop personalised treatment plans.

We previously conducted a series of studies on the value of dynamic ^18^F-FDG PET/CT in differential lung cancer diagnosis and N-staging (7–9). Our previous results have shown that, the dynamic metabolic parameter K_i_ has better differential diagnostic efficacy in the differential diagnosis of lung cancer, N-staging and prediction of EGFR status (K_i_ cut-off values, respectively: 0.0250 ml/g/min, 0.0220 ml/g/min, and 0.0350 ml/g/min), in particular, it improves specificity (7, 8). And, our study showed that, the average K_i_ in the lung malignant group (0.0267 ml/g/min) and metastatic lymph group (0.0190 ml/g/min) were higher than those in the lung benign group (0.0102 ml/g/min, *P*<0.01) and non-metastatic lymph group (0.0160 ml/g/min, *P=*0.01) (7, 8). In this case, we also further analysed the K_i_ values of the primary lung lesion, FDG-avid LNs, and subpleural nodes in the upper lobe of the left lung. Based on our previous research (7–9), we found that the K_i_ value of the primary lung foci (0.0668 ml/g/min) was significantly higher than the K_i_ value of the malignant lung group in our previous study (K_i_ cut-off value was 0.0250 ml/g/min), indicating the malignant nature of the disease. Among the mediastinal FDG-avid LNs, we measured the K_i_ value of a mediastinal region 4R (0.0650 ml/g/min), which was also significantly higher than the K_i_ value (K_i_ cut-off value was 0.0220 ml/g/min) of the metastatic LN in our previous study, also suggesting that this LN is a metastatic LN. In addition, we also measured the K_i_ of the nodules in the upper lobe of the left lung to be 0.0183 ml/g/min. Although we did not investigate the dynamic parameters of the metastatic lung lesions, the K_i_ trend also suggests malignancy of the nodule in the light of our previous experience in this area.

In terms of pathological features, tumors in the lungs characterized by small round cells with a sarcomatous morphology should be considered for undifferentiated small round cell sarcoma, lymphohematopoietic system tumors, and germ cell tumors. Therefore, panCK, CK8/18, VIM, CD20, CD3, CD99, CD34, CD117, MPO, SALL4 and BRG1 immunohistochemical staining tests were performed. The results showed that VIM exhibited punctate positive expression around the cell nucleus, and NUT exhibited positive expression in the cell nucleus. Therefore, a sarcoma with NUTM1 rearrangement was considered. Further FISH testing was conducted, which demonstrated separation of the NUTM1 gene. Thus, the pathological diagnosis was NUTM1 rearrangement sarcoma.

This case demonstrates the characteristics of NUTM1-rearranged lung sarcomas on ^18^F-FDG PET/CT and highlights the value of this imaging technique in the differential diagnosis and staging of malignant tumours. In the future, further attention will be paid to the mining of imaging features of NUTM1-rearranged lung sarcoma and the value of each metabolic parameter in differential diagnosis, staging, efficacy assessment and prognostic evaluation.

## 4 Conclusions

This case demonstrates the features of NUTM1-rearranged lung sarcomas on ^18^F-FDG PET/CT and highlights the value of this imaging technique in the differential diagnosis and staging of malignant tumours. The value of static and dynamic metabolic parameters in the differential diagnosis, staging, efficacy and prognostic assessment of NUTM1-rearranged lung sarcoma needs to be explored by collecting more cases.

## Data Availability

The original contributions presented in the study are included in the article/supplementary material, further inquiries can be directed to the corresponding author/s.
